# Risk assessment of upper limbs repetitive movements in a fish industry

**DOI:** 10.1186/s13104-019-4392-z

**Published:** 2019-06-24

**Authors:** Graziana Intranuovo, Luigi De Maria, Francesco Facchini, Armenise Giustiniano, Antonio Caputi, Francesco Birtolo, Luigi Vimercati

**Affiliations:** 1 Interdisciplinary Department of Medicine, Occupational Medicine “B. Ramazzini”, University of Bari Medical School, 70124 Bari, Italy; 2 Department of Mechanics, Mathematics and Management, Polytechnic University of Bari, Viale Japigia 182, 70123 Bari, Italy

**Keywords:** Fish industry, Checklist, Risk assessment, Work-related Musculoskeletal Disorders, Ergonomic risk

## Abstract

**Objective:**

Work-related Musculoskeletal Disorders (WMSDs) have a multifactorial origin: work-related risk factors and individual factors (age, sex, anthropometric characteristics). The purpose of the current study was the risk assessment of upper limb-WMSDs of workers engaged in tasks of anchovies filleting and packaging in a fish industry considering the ergonomic evaluation and the painful symptomatology complained by employees of different age. The activities were analysed by the American Conference Governmental Industrial Hygienists (ACGIH) method, the Strain Index (SI) method, the Rapid Upper Limb Assessment (RULA) method as well as the Occupational Rapid Assessment (OCRA) checklist. Workers answered the Italian version of the Nordic Musculoskeletal Questionnaire (NMQ).

**Results:**

The ACGIH method showed that packaging needs greater protection, while filleting requires ergonomic interventions. The SI showed a significant increasing risk for both tasks. The final score identified by RULA, for tasks of fish filleting and packaging, suggested a medium level of action, therefore were required additional observations. By OCRA checklist the final score for both tasks denoted a high risk.

## Introduction

Work-related Musculoskeletal Disorders (WMSDs) have become for some years subject of increasing interest in the safety and prevention at the workplace.

Most of symptoms and effects on work performance related to WMSDs are associated with upper limb disorders. Specific risk factors included are: repetitiveness of actions, use of force, awkward postures, lack of recovery periods, especially when such factors are present with one another [1–3].

There are other general risk factors. In particular, the environmental pollution is a public health problem, since many pollutants are identified as carcinogens of class I (Polycyclic Aromatic Hydrocarbons, PAHs; formaldehyde) according to International Agency for Research on Cancer (IARC) classification and have effects on the respiratory system [4–8]. In fact, it was demonstrated an association between an increased susceptibility to bone loss, therefore to MSDs, and exposure to inhalant organic dust, such as in the agriculture sector [9]. Another environmental risk factor for developing WMSDs is the impact of PAHs on chondrogenesis impairment [10].

This paper focuses on the evaluation of biomechanical overload risk of workers employed in a fish industry, located in Apulia Region (Southern Italy).

## Main text

### Materials and methods

The fish industry examined produces and commercializes semi-preserved and marinated blue fish.

This paper analyses two tasks: fish filleting and packaging, that cause a daily exposure to the risk factor “repeatability”, which consists in repeating the same motion for more than two times per minute for at least 2 h in the work shift. The study consists of workstation analysis performed both on site and, later, on recordings made during the inspections.

It has been considered an 8-h work shift with a 10-min break every 2 h. The cycle time to complete the packaging of a plastic pack is 70 s; that to fillet 42 fishes is 68 s.

After a careful video analysis, the following packaging actions have been calculated separately for each upper limb:

- to take the plastic pack: 3 times with the right hand; the operator places the plastic pack in front of him, then he places the full pack on the scale and if it has reached the desired weight, he will position it on the side;
- to take the fish: 30 times in which mainly uses the left limb (27 times with the left, 3 times with the right);
- to stretch it: 26 times using both hands;
- to put it into the plastic pack: 35 times;
- to press it (to create space): 11 times pressed mainly with the fingers of the right hand;
- to remove unsuitable parts: 3 times removed with the right hand.

The following filleting actions have been calculated separately for each upper limb:

- to take the fish: 44 times using mainly the right hand and 28 the left;
- to fillet it: the filleting action takes place with both hands, with one hand holding the fish and with the other removing the skin;
- to throw it into the water: 42 times with the left hand;
- to throw the skin into the trash: 44 times with the right hand.

During the activity of filleting the operators are standing, while during packaging are sitting with their back in anterior flexion. If on one hand, the sitting position reduces the physical fatigue, ensuring body stability and allowing the execution of precision movements; on the other hand, this position limits the workspace, reduces the use of force and increases the risk of postural fixity. The operators are sitting on stools without backrest and armrests. The operations require a reasonable accuracy and are made by the exclusive use of both arms, on metal tables with footrest.

The American Conference Governmental Industrial Hygienists (ACGIH) method, the Strain Index (SI) method, the Rapid Upper Limb Assessment (RULA) method and the Occupational Rapid Assessment (OCRA) checklist were adopted.

### The ACGIH method

The frequency of action has been calculated as the number of actions performed in 70 s.

For packaging, right limb frequency was 81/70 = 1.157 actions/s; left limb frequency was 78/70 = 1.114 actions/s. For filleting, the first was 114/68 = 1.676; the second was 133/68 = 1.956.

The Duty Cycle represents the percentage distribution of periods of work and recovery within the repetitive work cycle and it has been assigned a score of 80–100% for both operations.

By integrating frequency and Duty Cycle it has been possible to identify the value of the Hand Activity Level (HAL), that is equal to 7 for both tasks.

For the packaging task, the Peak of Force (PF) score was 2 (Weak); for filleting, PF score was 3 (Moderate).

For packaging, HAL = 7 and PF = 2, therefore the threshold limit value (TLV) diagram falls into a transition area (in yellow between the solid line and the dotted line corresponding to the action limit) designed to provide greater protection.

For filleting, HAL = 7 and PF = 3, then the TLV graph falls into the non-acceptability area that requires ergonomic interventions, therefore WMSD risk is greater for filleting task.

### The SI method

It has been assigned a light intensity for both tasks; duration of the exertion has been considered between 10 and 29 for packaging, 30–39 for filleting; right limb frequency = 81/1.1667 = 69.42 actions/min for packaging and 114/1.133 = 100.6 actions/min for filleting; left limb frequency = 78/1.1667 = 66.8 actions/min for packaging and 133/1.133 = 117.38 actions/min for filleting; the hand/wrist posture has been evaluated as bad and speed of work as fast for both tasks.

Multiplying the values for each variable, the SI is 13.5 for packaging, which corresponds to “Hazardous” that means it requires ergonomic interventions; the SI for filleting is 20.25, which means a higher increasing risk than packaging.

### The RULA method

The final score obtained by RULA method was 4 for both tasks, indicated a medium level of action (yellow band), therefore were required additional observations.

### The OCRA checklist

By applying OCRA checklist the final score was 29 for filleting task and 25 for packaging task, therefore it was higher than 22.6 for both tasks, thus denoting a high risk (purple band), in particular a higher risk in filleting employees.

Logistic regression shows a greater painful symptomatology among filleting employees, also by age and task duration adjusting, but this result is not statistically significant.

### The NMQ

Finally, workers answered a self-administered questionnaire, which is the Italian version of Nordic Musculoskeletal Questionnaire (NMQ) standardized for the analysis of musculoskeletal symptoms and functional impairments.

The industry employed in filleting and packaging tasks is composed by 60 workers totally. Among them, 50 subjects (47 women and 3 men) participated to this study, then there was an adhesion rate of about 83.3%. Two of 50 workers were excluded as missing subjects because they did not answer completely to NMQ, so it was not possible to categorize them for task. Exclusion criteria were low back pain and serious disorders in the nine body regions examined, existing prior to the date of recruitment. However, no one was suffering of such pre-existing disorders.

A database was made with information of NMQ about age, duration and kind of task, hours worked weekly and referred pain to nine body regions.

Workers were divided into three task categories: 28 workers assigned to filleting, 7 to filleting and packaging, 13 to packaging (Table 1). There are three age categories: 15 workers (5 filleting, 5 filleting/packaging, 5 packaging) are less than 30, 12 workers (8 filleting, 1 filleting/packaging, 3 packaging) are between 30 and 39, 21 workers (15 filleting, 1 filleting/packaging, 5 packaging) are 40 or more than 40 (Tables 1, 2). Workers were divided also into three categories for duration of task: 25 workers (14 filleting, 5 filleting/packaging, 6 packaging) are working for less than 2 years, 8 (6 filleting, 1 filleting/packaging, 1 packaging) for 2 or more than 2 years but less than 4 years, 15 (8 filleting, 1 filleting/packaging, 6 packaging) for 4 years or more than 4 years (Tables 1, 3).

**Table 1:** Correlation between referred pain and task, pain and age, pain and duration of task

Pain	Task			Age [years]			Duration of task [years]		
	Filleting	Filleting/packaging	Packaging	< 30	30/39	≥ 40	< 2	2/4	≥ 4
Neck	17	3	11	7	9	15	13	8	10
Shoulders	20	4	12	11	10	15	20	7	9
Elbows	3	0	2	0	0	5	1	1	3
Wrists/hands	17	5	11	11	6	16	15	7	11
Dorsal region	13	2	8	7	4	12	11	6	6
Lumbar region	12	2	7	6	5	10	10	5	6
Hips/thighs	3	0	3	1	2	3	2	1	3
Knees	8	1	4	4	2	7	6	3	4
Ankles/feet	6	0	5	2	3	6	3	2	6
Total	28	7	13	15	12	21	25	8	15
Neck	60.7%	42.9%	*84.6*%	46.7%	*75*%	71.4%	52%	*100*%	66.7%
Shoulders	71.4%	57.1%	*92.3*%	73.3%	*83.3*%	71.4%	80%	*87.5*%	60%
Elbows	10.7%	0%	*15.4*%	0%	0%	*23.8*%	4%	12.5%	*20*%
Wrists/hands	60.7%	71.4%	*84.6*%	*73.3*%	50%	*76.2*%	60%	*87.5*%	73.3%
Dorsal region	46.4%	28.6%	*61.5*%	*46.7*%	33.3%	*57.1*%	44%	*75*%	40%
Lumbar region	42.9%	28.6%	*53.9*%	40%	41.7%	*47.6*%	40%	*62.5*%	*40*%
Hips/thighs	10.7%	0%	*23.1*%	6.7%	*16.7*%	14.3%	8%	12.5%	*20*%
Knees	28.6%	14.3%	*30.8*%	*26.7*%	16.7%	*33.3*%	24%	*37.5*%	26.7%
Ankles/feet	21.4%	0%	*38.5*%	13.3%	25%	*28.6*%	12%	25%	*40*%
Average	39.3%	27%	*53.9*%	36.3%	38%	*47.1*%	36%	*55.6*%	*43*%

In italic are categories that complain most of painful symptoms, in particular it was calculated the average of workers who complain of symptoms for each category

**Table 2:** Referred pain in relation to task and age

Task	Age [years]	Pain									
		Neck	Shoulders	Elbows	Wrists/hands	Dorsal Region	Lumbar Region	Hips/thighs	Knees	Ankles/feet	Total
Filleting	< 30	2	3	0	3	3	2	0	1	1	5
	30/39	5	6	0	3	2	2	0	2	1	8
	≥ 40	10	11	3	11	8	8	3	5	4	15
Filleting/packaging	< 30	2	3	0	4	1	1	0	1	0	5
	30/39	1	1	0	1	1	1	0	0	0	1
	≥ 40	1	0	0	0	0	0	0	0	0	1
Packaging	< 30	3	5	0	4	3	3	1	2	1	5
	30/39	3	3	0	2	1	2	2	0	2	3
	≥ 40	4	4	2	5	4	2	0	2	2	5
Filleting	< 30	40%	60%	0%	60%	*60*%	*40*%	0%	20%	20%	5
	30/39	62.5%	*75*%	0%	37.5%	25%	25%	0%	25%	12.5%	8
	≥ 40	*66.7*%	*73.3*%	*20*%	*73.3*%	*53.3*%	*53.3*%	*20*%	*33.3*%	*26.7*%	15
Filleting/packaging	< 30	40%	60%	0%	80%	20%	20%	0%	20%	0%	5
	30/39	100%	100%	0%	100%	100%	100%	0%	0%	0%	1
	≥ 40	100%	0%	0%	0%	0%	0%	0%	0%	0%	1
Packaging	< 30	60%	*100*%	0%	*80*%	*60*%	60%	20%	*40*%	20%	5
	30/39	*100*%	*100*%	0%	66.7%	33.3%	*66.7*%	*66.7*%	0%	*66.7*%	3
	≥ 40	*100*%	80%	*40*%	*100*%	*80*%	40%	0%	*40*%	40%	5

In italic are categories that complain most of painful symptoms

**Table 3:** Referred pain in relation to task and duration of task

Task	Duration of task [years]	Pain									
		Neck	Shoulders	Elbows	Wrists/hands	Dorsal Region	Lumbar Region	Hips/thighs	Knees	Ankles/feet	Total
Filleting	< 2	7	12	1	8	7	7	1	4	1	14
	2/4	6	5	1	5	4	3	1	3	2	6
	≥ 4	4	3	1	4	2	2	1	1	3	8
filleting/packaging	< 2	2	2	0	3	0	0	0	1	0	5
	2/4	1	1	0	1	1	1	0	0	0	1
	≥ 4	0	1	0	1	1	1	0	0	0	1
Packaging	< 2	4	6	0	4	4	3	1	1	2	6
	2/4	1	1	0	1	1	1	0	0	0	1
	≥ 4	6	5	2	6	3	3	2	3	3	6
Filleting	< 2	50%	*85.7*%	7.1%	57.1%	50%	*50*%	7.1%	28.6%	7.1%	14
	2/4	*100*%	*83.3*%	*16.7*%	*83.3*%	*66.7*%	*50*%	*16.7*%	*50*%	*33.3*%	6
	≥ 4	50%	37.5%	12.5%	50%	25%	25%	*12.5*%	12.5%	*37.5*%	8
Filleting/packaging	< 2	40%	40%	0%	60%	0%	0%	0%	20%	0%	5
	2/4	100%	100%	0%	100%	100%	100%	0%	0%	0%	1
	≥ 4	0%	100%	0%	100%	100%	100%	0%	0%	0%	1
Packaging	< 2	66.7%	*100*%	0%	66.7%	*66.7*%	*50*%	16.7%	16.7%	33.3%	6
	2/4	100%	100%	0%	100%	100%	100%	0%	0%	0%	1
	≥ 4	*100*%	83.3%	*33.3*%	*100*%	50%	*50*%	*33.3*%	*50*%	*50*%	6

In italic are categories that complain most of painful symptoms

## Results

On a first assessment, workers assigned to packaging complained of more pain to the various body segments. There was no apparent difference in the symptoms complained by workers belonging to the three age categories (< 30 years, 30–39 years, ≥ 40 years). On the other hand, workers employed for more over 2 years complained of more pain to the various body regions, than workers employed for less than 2 years (Table 1).

On a second assessment, workers assigned to filleting, who were more over 40 years old, complained of more symptoms to the various body regions, than younger workers; the only exception is the back pain complained also by younger workers assigned to filleting task. For packaging, workers complained of symptoms regardless of their age category (Table 2).

Workers employed for more over 2 years complained of more pain to the various body regions, than workers employed for less than 2 years, only for packaging task, less for workers assigned to the filleting task (Table 3).

A link between complaints and workplace assessments was evaluated. In particular, filleting operators have four standing workplaces, while packaging operators have four sitting workplaces. Filleting task was found more dangerous than packaging, probably due to the standing position of filleting employees and because they perform more times each action, than packaging employees who are sitting and make less times each action. Furthermore, filleting employees perform less kinds of actions than packaging workers.

It would be interesting the correlation between ergonomic aids (e.g. ergonomic stool with backrest, armrest; wrist supports; adjustment options to coordinate with workspace needs) and different ergonomic assessment methods used. However, work tasks analysed in this paper are very elementary, therefore there are not any ergonomic aids such that they would lead to a change in the conclusions set out by the different ergonomic assessment methods.

## Discussion

Despite most workers employed in filleting task are older, in particular 23 on 28 are more than 30, and filleting task is more dangerous for worker’s health than packaging task according to the results of OCRA checklist, filleting employees complain of less symptoms than packaging employees (Table 2).

However, most filleting employees, in particular 20 on 28, have been working for less than 4 years, while only about the half of packaging employees, 6 on 13, have been working for less than 4 years.

It results that the most important risk factor in determining painful symptomatology in the various body regions is not the age, but the duration of task, though filleting task predisposes more to develop painful symptoms according to OCRA checklist.

In literature, a positive correlation between complaints and years of work seniority was found among medical staff carrying out endoscopy activity, also between complaints and number of examinations performed and non-ergonomic workplace [11].

Lorusso et al. showed a high prevalence of musculoskeletal disorders among Italian X-ray technology students, whose tasks frequently involve manual handling of patients and materials [12].

A solution to the problem of work-related biomechanical overload is the implementation of a job rotation program. However, it was observed that in the long run, job rotation can also increase overload on other body regions, for example low back pain, because probably there is workers’ exposure to the same risk factors, even when they switch task [13].

It also could be necessary to identify the best solution of job rotation in presence of a sub-group of operators requiring lower risk exposure [14].

The application of ergonomic criterions also in equipment manufacturing, as well as in material handling activities, could minimize the impact of WMSDs [15, 16].

## Limitations

In the present study, we have no information about some complementary factors, such as vibration and extreme conditions of temperature.

In addition, authors are aware that the power of the study could have been higher with a larger sample size and that some distortions may occur due to the prevalence type of data.

## Data Availability

Data will be provided upon request to Luigi Vimercati (MD, PhD), Interdisciplinary Department of Medicine, Occupational Medicine “B. Ramazzini”, University of Bari Medical School, 70124 Bari, Italy. E-mail: luigi.vimercati@uniba.it

## Abbreviations

WMSDs: Work-related Musculoskeletal Disorders
ACGIH: American Conference Governmental Industrial Hygienists
RULA: Rapid Upper Limb Assessment
OCRA: Occupational Rapid Assessment
NMQ: Nordic Musculoskeletal Questionnaire
PAHs: Polycyclic Aromatic Hydrocarbons
IARC: International Agency for Research on Cancer
HAL: Hand Activity Level
PF: Peak of Force
TLV: threshold limit value
IEM: Intensity of Exertion Multiplier
DEM: Duration of Exertion Multiplier
EMM: Exertions per Minute Multiplier
HPM: Hand/wrist Posture Multiplier
SWM: Speed of Work Multiplier
DDM: Duration per Day Multiplier
